# Evaluation of cardiovascular autonomic dysfunction according to heart rate turbulence and variability in patients with relapsing remitting multiple sclerosis

**DOI:** 10.3906/sag-1912-6

**Published:** 2020-04-09

**Authors:** Serkan GÖKASLAN, Hayri DEMİRBAŞ, Ciğdem ÖZER GÖKASLAN

**Affiliations:** 1 Department of Cardiology, Faculty of Medicine, Afyonkarahisar University of Health Sciences, Afyonkarahisar Turkey; 2 Department of Neurology, Faculty of Medicine, Afyonkarahisar University of Health Sciences, Afyonkarahisar Turkey; 3 Department of Radiology, Faculty of Medicine, Afyonkarahisar University of Health Sciences, Afyonkarahisar Turkey

**Keywords:** Multiple sclerosis, arrhythmias, autonomic nervous system disorders

## Abstract

**Background/aim:**

Multiple sclerosis (MS) is an autoimmune disease characterized by neurodegeneration or demyelination; the relapsing–remitting phase of MS is characterized by acute exacerbation of disease activity. The most commonly used noninvasive approach to assess autonomic function is the determination of heart rate turbulence (HRT) and heart rate variability (HRV). The aim of this study was to evaluate the presence of cardiovascular autonomic dysfunction using HRT and HRV parameters determined via 24-h Holter ECG monitoring in patients with relapsing–remitting MS without known heart disease.

**Materials and methods:**

The study included 26 patients diagnosed with relapsing–remitting MS and 22 age- and sex-matched healthy controls. HRT and HRV parameters were analyzed via 24-h Holter ECG monitoring. Magnetic resonance imaging findings were reevaluated to identify any demyelinating lesions in the brain stem.

**Results:**

The HRV parameters of SDNNI (mean of the standard deviations of all normal sinus RR intervals in all 5-min segments), rMSSD (root–mean–square successive difference), and sNN50 (percentage of successive normal sinus RR intervals >50 ms) were significantly lower in the MS group than in the control group (P < 0.05).

**Conclusion:**

This study revealed that the patients with MS had reduced HRV; this was demonstrated by dysfunction with regard to parasympathetic and sympathetic parameters in HRV analysis.

## 1. Introduction

Multiple sclerosis (MS) is an autoimmune disease that causes neurodegeneration or demyelination caused by the entrance of autoreactive lymphocytes into the central nervous system (CNS). Although the etiology of MS remains unknown, environmental and genetic factors are thought to contribute to its development. Multiple sclerosis (MS) is a rare disease, characterized by a chronic course with intermittent relapses According to WHO, the disease burden of MS accounts for 0.1% of total disease burden of neurologic diseases [1–3]. The course of MS is explained broadly with two phases: the first phase is the relapsing–remitting phase, which is characterized by acute exacerbation of disease activity and is pathologically associated with CNS inflammation. The second phase (progressive) involves slow but constant progression of neurological deficits due to degeneration of the CNS [1]. MS usually begins in the form of the relapsing–remitting phase, but a few cases may present with the progressive form at disease onset. The number of relapses, their frequency, and the development of demyelinating lesions appear to be predictors of disease progression [4].

Although it is known that autonomic dysfunction might occur in organs with autonomic nervous system (ANS) innervation, there are some studies on autonomic dysfunction in MS. Furthermore, patients with MS might be completely asymptomatic, particularly in the steady state, and thus may require active examination of autonomic dysfunction through ANS testing [5]. The cardiovascular autonomic system is currently the most comprehensively researched part of the ANS in patients with MS, quite possibly due to the ease and reliability of testing, and also the fact that brain stem lesions may lead to cardiovascular autonomic dysfunction [6]. The locus coeruleus is located in the brain stem and is responsible for the synthesis of norepinephrine. Damage to the locus coeruleus might cause sympathetic dysfunction in MS [7]. Although the parasympathetic system is reportedly preserved in the initial disease period, as the disease duration is prolonged, parasympathetic dysfunction may also be observed [8].

The most common noninvasive methods used to assess autonomic function are evaluations of heart rate turbulence (HRT) and heart rate variability (HRV) with 24-h Holter electrocardiography (ECG) monitoring. HRT refers to physiological short-term baroreflex-regulated fluctuations in sinus rhythm following premature ventricular contraction (PVC) [9]. The fluctuation begins with heart rate acceleration for a short time and is followed by a slow decrease through mediation by the baroreflex; it usually comprises 15–20 consecutive beats [10]. The following parameters are used to evaluate HRT: (1) turbulence onset (TO), which reflects the initial heart rate increase following a ventricular premature beat, and (2) turbulence slope (TS), which reflects heart rate deceleration [11,12].

Similar to HRT, HRV also provides important data regarding cardiac autonomic function [13]. HRV is based on the evaluation of RR intervals (the intervals between successive heartbeats), and it corresponds to cyclic fluctuations in resting heart function. Increased sympathetic activity and withdrawal of vagal tone lead to a decrease in HRV, which is believed to be a mechanism for the development of life-threatening arrhythmias [13,14].

The aim of this study was to evaluate the presence of cardiovascular autonomic dysfunction using HRT and HRV parameters determined with 24-h Holter ECG monitoring in patients with MS but no known heart disease. 

## 2. Materials and methods

### 2.1. Study design and patient selection

This study was conducted from May 2015 to May 2018 and included 26 patients diagnosed with MS and 22 volunteers (controls) without any known cardiovascular diseases who were similar to the patients in terms of age and sex. Holter ECG monitoring was performed in the patients included in the study because of palpitation symptoms; there was no drug use that would affect HRT and HRV measurements. Informed consent was obtained from all participants, and the study was approved by the local ethics committee.

All patients with MS included in this study were in the relapsing–remitting phase according to the McDonald criteria [15], and none of the patients had the progressive course of the disease. All the patients had been diagnosed with MS within the last 3 years. Additionally, all patients had sinus rhythm, and none of the patients had any known cardiovascular disease.

Patients with myocardial infarction, moderate or severe heart valve diseases, heart rhythm disorders including atrial fibrillation, congenital heart diseases, diabetes mellitus, hypertension, or cerebrovascular events, and those using medications that could affect the autonomic nervous system (anticholinergics, antiarrhythmics, sympathomimetics, parasympathomimetics, etc.) were excluded from the study. 

Serum electrolyte values, atrial conduction times measured in ECG, and echocardiography parameters were normal in all patients and the control group.

### 2.2. Measurements

In all patients and controls, 24-h Holter ECG monitoring was applied (Reynolds Medical Pathfinder Software Version V8.255; Reynolds Medical, Hertford, UK). Holter ECG monitoring was applied within 3 days after performing brain magnetic resonance imaging (MRI) in the patient group. The Holter records were examined. Artefacts were excluded manually. As HRT data cannot be obtained in patients without a PVC, these patients were excluded from the study.

The HRT parameters (TO and TS) were calculated by using a computer program (HRT View Software Version 0.60–0.1, Munich, Germany). TO was expressed as the percentage difference between 2 RR intervals measured immediately after a VPB and immediately before a VPB. The TS value was defined and calculated as the maximum positive slope of a regression curve obtained from any sequence of 5 subsequent RR intervals within the first 20 sinus rhythm intervals after a VPB. The TO value was calculated for each VPB separately and the final data was defined as the mean values of these separate measurements. A TO value of ≥0% and a TS value of ≤2.5 ms/RR were considered abnormal [16].

HRV analysis was performed according to the guidelines of the European Society of Cardiology and the North American Society of Pacing and Electrophysiology in all participants, with HRV measurements from 24-h Holter ECG records [14]. Evaluation of HRV were performed by using a computer program (Cardioscan 12.0, DMS, Ventura, CA, USA). The nocturnal 6-h part was determined following the evident day–night switch in the length of RR intervals. The HRV measurements included the standard deviation of all normal sinus RR intervals over 24 h (SDNN), mean of the standard deviations of all normal sinus RR intervals for all 5-min segments (SDNNI), standard deviation of the averaged normal sinus RR intervals for all 5-min segments (SDANN), root–mean–square of the successive normal sinus RR interval difference (rMSSD), and percentage of successive normal sinus RR intervals >50 ms (sNN50).

MRI findings of the patients were reevaluated to identify any demyelinating lesions in the brain stem. The results were noted as presence/absence of lesions in the brain stem.

### 2.3. Statistical analysis

All analyses were performed using SPSS software, version 24 (IBM Corp., Armonk, NY, USA). A P-value of <0.05 was considered significant. The distributions of HRT and HRV parameters in the MS and control groups were examined using the Shapiro–Wilk test. Continuous data that did not show normal distribution were compared using the Mann–Whitney U test. Categorical parameters were evaluated and compared with Chi-square tests. HRT and HRV values were adjusted according to age by analysis of covariance. For descriptive statistics, frequencies were used for categorical data and median and interquartile ranges were used for quantitative data not normally distributed. Correlation analyses were performed using Spearman’s rank correlation coefficients. A post-hoc power analysis was performed to confirm that the current study had adequate power (99%).

## 3. Results

Of the 26 patients with MS, 6 were male and 20 were female, and the mean patient age was 43.5 ± 10.7 years (Table 1). In this group, the mean disease duration after diagnosis at the time of the study was 2.3 ± 0.6 years. There was no significant difference in age or sex between the MS and control groups. Additionally, there was no difference in the mean heart rate between the 2 groups. Conversely, the HRV parameters of SDNNI, rMSSD, and sNN50 were significantly lower in the MS group than in the control group (P = 0.009, P = 0.011, and P = 0.041, respectively). However, there were no differences in the other HRV parameters (SDNN and SDANN) and the HRT parameters (TO and TS) between the 2 groups (P = 0.373, P = 0.454, P = 0.135, and P = 0.375, respectively) (Table 2).

**Table 1:** Demographic characteristics of the patients with MS and controls.

	Patients with MS (n = 26)	Controls (n = 22)	P-value
Age (years) (mean ± SD)	43.5 ± 10.7	41.7 ± 9.4	0.311
Sex (number of females)	20 (76.9%)	14 (63.6%)	0.169

MS = multiple sclerosis; SD = standard deviation.

**Table 2:** HRT and HRV parameters of the patients with MS and controls.

		Patients with MS(n = 26)	Controls(n = 22)	P-value
HRT	TO	–0.02 (0.03)	–0.02 (0.05)	0.373
	TS	8.04 (13.51)	10.83 (9.34)	0.454
HRV	SDNNI	41.00 (18.25)	51.00 (20.50)	0.009*
	SDNN	124.50 (51.25)	137.00 (59.25)	0.135
	SDANN	117.00 (41.00)	127.50 (60.00)	0.375
	rMSSD	20.50 (13.75)	29.50 (20.50)	0.011*
	sNN50	2357.50 (6948.25)	5865.50 (12,981.20)	0.041*

Data are presented as median (interquartile range). HRT = heart rate turbulence; HRV = heart rate variability; MS = multiple sclerosis, TO = turbulence onset; TS = turbulence slope; SDNN = Standard deviation of NN intervals; SDNNI = SDNN index; SDANN = Standard deviation of the average NN intervals for each 5-min segment; rMSSD = Root–mean–square of successive RR interval differences; sNN50 = Percentage of successive RR intervals that differ by more than 50 ms. *P < 0.05.

There were significant negative correlations between age and all HRV parameters (Table 3). Furthermore, there were significant differences in SDNNI, rMSSD, and sNN50 between the 2 groups after adjusting the variables according to age through analyses of covariance. There were no significant differences in the nocturnal HRV parameters between the 2 groups (P = 0.154). 

**Table 3:** Correlations of HRV parameters with age.

		r	P
	SDNN	–0.386	0.007
	SDNNİ	–0.505	<0.001
HRV	SDANN	–0.328	0.023
	rMSSD	–0.410	0.004
	sNN50	–0.504	<0.001

*Spearman’s correlation analysis.

Brain MRI findings revealed brain stem lesions in 13 patients and no brain stem lesions in the remaining 13 patients. There were no significant differences in the HRT and HRV parameters between patients with and those without brain stem lesions.

## 4. Discussion

Autonomic dysfunction is an important symptom as well as a factor for morbidity among patients with MS. The limited clinical use of autonomic dysfunction testing methods in patients with MS makes assessment difficult. However, the details of these dysfunctions can shed new light on the management of the disease. In the absence of reliable methods of measurement for other parameters, the determination of cardiovascular autonomic dysfunction using parameters such as HRV and HRT has received interest [17].

HRV primarily measures tonic vagal activity [18]. Under resting conditions, vagal tone is predominant but vagal and sympathetic activities are in constant balance. Reduced HRV is most commonly seen in patients with heart failure, diabetic neuropathy, or myocardial infarction [13]. 

In the present study, the HRV parameters of SDNNI, rMSSD, and sNN50 were significantly lower in the MS group than in the control group. These results indicate autonomic imbalance in patients with MS. However, there were no significant differences in other HRV parameters (i.e. SDNN and SDANN) between the groups. A previous study reported that the value of HRV parameters decreased as patients with MS aged [19]. In another study, parasympathetic activity was reported to be lower in patients who had been living with MS for more than 5 years compared to those who had been diagnosed within the last 5 years [20]. In the present study, all patients had been diagnosed with MS within the last 3 years, and HRV parameters significantly decreased as patient age increased. This finding supports the theory that parasympathetic activity decreases and sympathetic activity becomes more dominant as patients age, regardless of disease duration, which may seem counterintuitive considering that MS is a cause of progressive neurodegeneration. However, in a very recent study that included 43 patients who had been newly diagnosed with MS, it was reported that 43% of the patients had autonomic dysfunction as measured by the Survey of Autonomic Symptoms [21]. Similarly, when the ANS symptoms of patients with long-standing MS (n = 33, mean disease duration: 97.6 ± 75.5 months) were assessed with a battery of tests, a similar frequency (45.5%) of autonomic abnormality was reported [22]. Even though patient groups were not very large (as is the case in most studies), these similarities suggest that the development of ANS problems may not be directly associated with disease duration (or even progression). In a seemingly conflicting opinion, Studer et al. suggested that sympathetic dysfunction was directly associated with disability progression in MS; however, they also reported that only patients with the progressive course of disease demonstrated such a relationship, while those with the relapsing–remitting course had no differences from healthy controls in terms of autonomic function [23]. The latter finding requires further analysis, as autonomic dysfunction was found to be similar to that of controls; but it is important to note that autonomic dysfunction was assessed with the head-up tilt test (causing stress) which may have prevented the detection of underlying dysfunction which may have been more accurately determined with 24-h Holter ECG.

Previous studies have reported reduced HRV in patients with MS in the presence of brain stem lesions, but this decrease was also associated with general disability, duration of the disease, and number of recurrences [24–26]. In the present study, brain MRI revealed brain stem lesions in half of the patients. In contrast to previous findings, there were no significant differences in HRT and HRV parameters between patients with and without brain stem lesions. This converse result might be due to short disease duration and the low recurrence rate observed in our group of patients. Furthermore, considering that older age significantly influences HRV values in healthy subjects, it is apparent that further studies should assess HRV with regard to age groups in patients with MS [27].

HRT impairment reflects cardiac autonomic dysfunction, particularly in association with impaired baroreflex sensitivity and decreased parasympathetic activity. Many previous studies have reported abnormal HRT in a variety of diseases, and have associated these findings with impaired ANS and baroreflex response [28–31]. Despite the presence of studies focused on baroreflex impairment in MS, to our knowledge, no study has investigated the relationship between HRT and baroreflex in patients with MS. The present study is the first to assess this relationship. Our results pertaining to the comparison of HRT between patients diagnosed with MS within the last 3 years and healthy controls revealed no significant differences between these groups. In contrast to the findings of previous studies reporting that the baroreflex is reduced in patients with MS [32,33], the present study found that the baroreflex is intact, at least during the initial years of the disease.

The most significant finding of this study was that parasympathetic cardiovascular tonus was significantly lower in patients with MS in the relapsing–remitting phase than in controls. There are contradictory reports on the presence and degree of cardiovascular autonomic dysfunction in patients with MS [23,34]. Adamec et al. found worse autonomic function in patients with a progressive course compared to those with relapsing–remitting course, even when corrections for age, sex, and disease duration were performed [35]. However, in an earlier crosssectional study by Flachenecker et al., it was found that 39% of patients with relapsing–remitting MS had abnormal sympathetic test results, while no abnormalities were found in any of the stable MS patients. Further, even though parasympathetic results were similar in these 2 groups at baseline, longitudinal analyses showed that patients demonstrated worsening of parasympathetic dysfunction, while sympathetic dysfunction was unchanged hence, supporting our findings [36]. Nevertheless, there are major differences among studies in the literature and these discrepancies may have resulted from various causes, including methodological differences, sample collection/evaluation, and the clinical state of the patients included in each study. We believe the main reason for conflicting results is that previous studies mostly did not consider the relapsing–remitting phase of the disease, whereas the present study only included patients in this phase.

The main limitation of our study was the absence of spectral HRV analysis. Consequently, only parasympathetic tonus was assessed and sympathetic tonus could not be evaluated. Another important limitation of our study is the absence of a gold-standard technique to determine autonomic activity: we assessed function through evaluation of HRT and HRV values, which may have yielded inconsistent results. However, considering that there is no method to determine autonomic function as a whole, it is evident that all studies in this field suffer from this limitation.

In conclusion, despite conflicting results, the present study has revealed that some HRV values were lower in patients with MS compared to healthy controls, as demonstrated by dysfunctional findings with regard to parasympathetic parameters in HRV analysis. Thus, patients in the relapsing–remitting phase of MS may have cardiovascular autonomic dysfunction, but it is also apparent that there are many contributors to the dysfunction of the autonomic system. The present findings need to be confirmed in further studies involving a greater number of patients who should be stratified with regard to duration with MS.

## Acknowledgments

We would like to thank Prof Dr. İsmet Doğan for his help in statistical analysis of our study.

## Informed Consent

Informed consent was obtained from all participants of the study.
